# A Procedure for the More Secure Detection of Arsenic in Minute Quantity

**Published:** 1849-01

**Authors:** T. F. Geoghegan

**Affiliations:** Prof. Forens. Med. Royal College of Surgeons in Ireland


### CHEMISTRY.

*A procedure for the more secure Detection of Arsenic in minute quantity.* By T. F. GEOGHEGAN, M. D., Prof. Forens. Med. Royal College of Surgeons in Ireland.—The practitioner when called upon in his capacity of medical witness to pronounce on the presence of poison, when the quantity capable of elimination is extremely minute, often experiences considerable difficulty in giving an opinion sufficiently definite for judical purposes.

The embarrassment alluded to does not arise from the organic contaminations of the matter examined, nor from the effects of putrefaction upon it. The former may no doubt completely mask the presence of vegetable poisons, while the latter may render nugatory a search for the mineral acids, or, in conjunction with the first-named influence, may so far modify some of the metallic poisons as practically to place them, in our reports, in an equivocal position, considered as deadly agents. The inconvenience which the present communication proposes to remove in a particular case, results from the impossibility of subdividing a very minute quantity of a poisonous substance, so as to apportion it amongst such a number of reagents as shall suffice for its *unequivocal* detection, without obscuring the indications of some, or multiplying those of others. It is presumed that most practitioners have encountered this difficulty in the case of arsenic—a substance which so frequently gives rise to grave and intricate medicolegal inquiries. The number of reagents which may be requisite to furnish this conjoint evidence for the valid discrimination of poison, can only be indicated by reference to individual substances, and by a careful consideration of the chemical habitudes of each. Even in special cases much difference of opinion and practice prevails. Thus, in relation to arsenic, it has been the custom to attach, as I conceive, too exclusive importance to reduction and oxidation, in comparative neglect of other indications, which, when properly associated, are equally, if not more, distinctive, and which I propose to show may, in almost all cases, be conjoined with the former. The method of reduction devised by Reinsch, has, in the case of complex mixtures, from its facility and delicacy, superseded all others, with the exception of that of Marsh. The deposition, therefore, of metallic arsenic upon copper, may be taken as the starting-point in our inquiry.

I deem it not unimportant to notice that I have sometimes encountered deposits on copper closely simulating in appearance that produced by arsenic, although not a particle of arsenious acid could be obtained by sublimation, nor any evidence of the presence of other metallic poison. On the other hand, the usual tin-grey metallic appearance of arsenic (when decisively precipitated upon copper) is often replaced by a black-coating quite destitute of metallic lustre.

With reference to the sublimate, afterwards obtained by heating the copper foil, I have to observe, that when its amount is very minute, although its apparently crystalline character can generally be recognised by a lens, or even by the naked eye, the precise figure of the crystal cannot be discerned with sufficient precision without the aid of a microscope of ordinary power. The octohedra are then observed with admirable definition, either perfect, or more frequently variously truncated on their terminal and base angles, and intermixed with a few tetrahedra.

Crusts, seemingly crystalline to the lens, are occasionally obtained from well-dried foil, apparently covered with arsenic, which on being submitted to the microscope prove to be either globules of fluid, or crystals not having the figure of arsenious acid, and devoid also of its chemical properties. The composition of the latter I have not as yet succeeded in determining. The globules of fluid are probably either water derived from a thin film of organic matter, which adheres, despite of washing and drying, to the copper (especially in operating on fluids previously submitted to the proccss of carbonization by sulphuric acid,) or hydrochloric acid, resulting from the partial decomposition of the sub-chloride of copper, which also attaches to the foil, and of which a portion sublimes as an amorphous crust, deposited in the tube beneath the true arsenical one.

I am satisfied from the foregoing considerations, that any who shall feel disposed to accept as evidence of arsenic a well-marked coating of the copper, together with the production of a brilliant, crystalline-looking sublimate, fall into a dangerous error. If, however, the crystalline *figures* above stated be observed with the microscope, theyare so peculiar, constant, and well-defined, as of themselves to furnish, under the circumstances of their production, a strong presumption of the presence of arsenious acid. It is true that the latter compound is dimorphous ; I believe, notwithstanding, that it is never obtained as a sublimate in medico-legal researches, in any but the octohedral form. Thus I have found that a solution of arsenite of ammonia yields, by spontaneous evaporation, silvery crystalline scales of arsenious acid, apparently similar to those described by Wöhler as referable to the rhombic system. On heating these they sublime in the ordinary form.*

Few, however, who are conversant with the grave impossibilities of public medicine, will be content with obtaining the amount of evidence already considered : and hence the chemical properties of a solution of the sublimate are generally sought to be scrutinized, but in many cases, from its very trivial amount, with but indifferent success. Minute quantities, doubtless, when dissolved, will furnish an indication by the use of a single test, as the ammonio-nitrate of silver, or sulphuretted hydrogen. It would be, however, much more satisfactory to obtain, if possible, the conjoint evidence of *all* the fluid tests. The necessity for a method fulfilling the above indication having frequently forced itself upon me in practice, I have been led to propose the following procedure, by which a given quantity of arsenious acid may be transferred undiminished to each of the fluid tests in succession. As minute precautions in manipulation vitally affect the result, it may be premised, that want of success in the application of the fluid-tests to small sublimates, sometimes arises from not reducing the latter to powder before attempting their solution. The sublimate being carefully detached by a glass rod, aided by a fine stream of distilled water, should be received in a small porcelain mortar, and carefully triturated. The solution having been effected by boiling, should (1) be precipitated when cool, by ammonio-nitrate of silver. The yellow arsenite obtained is to be next decomposed by a slight excess of pure hydrochloric acid, and the filtered solution treated (2) by a current of sulphuretted hydrogen. Having ascertained the solubility of the resulting sulphuret in ammonia, it should now be dissolved in nitro-muriatic acid, and evaporated to dryness (avoiding excess of heat at the close,) redissolved and precipitated (3) by nitrate (or ammonio-nitrate) of silver, which yields the brick-red arseniate (4.) Finally, the latter being decomposed by hydrochloric acid, in minimum quantity, the filtrate should be heated with a few drops of an aqueous solution of sulphurous acid, the excess of the latter expelled, and hydrated oxide of copper, with ammonia, in minute quantity, added. We can thus elicit the reactions of the *four* fluid tests from a quantity of arsenious acid which would prove refractory by the common method of subdivision, and are hence enabled to ensure a satisfactory issue in difficult cases. The final step of the operation is not always successful, the ammonio-sulphate of copper being, even in experiments in larger quantities, a much less delicate test than those previously named. Having obtained, however, the antecedent results, the evidence of the presence of arsenic may be deemed complete. Modifications of the foregoing method will at once suggest themselves, and may be adopted at pleasure. It may occur to the instructed reader, that the success of the

* It is known that arsenite of ammonia cannot be obtained by evaporating its solution in the ordinary way ; and I have ascertained, by experiment, that the above crystals, from *spontaneous* evaporation, are destitute of ammonia, and have a *faint* acid reaction like that of opaque arsenic. The transparent acid will be found (as stated by Bussy) at *once* to redden litmus.

copper test might be secured by reprecipitation and sublimation of the arsenic subsequent to the formation of arseniate of silver ; as, however, there is reason to believe that no inconsiderable portion of the metal is often retained by the copper foil as an arseniuret, this procedure cannot be recommended. Such retention indeed constitutes a reason for assigning to the method of Marsh a superior delicacy, although, in a practical point of view, that of Reinsch is equal to all the emergencies of medico-legal experience.—*Med. Gaz.*

